# Differential Expression of Wound Fibrotic Factors between Facial and Trunk Dermal Fibroblasts

**DOI:** 10.3109/03008207.2012.657309

**Published:** 2012-07-24

**Authors:** Masakazu Kurita, Mutsumi Okazaki, Akiko Kaminishi-Tanikawa, Mamoru Niikura, Akihiko Takushima, Kiyonori Harii

**Affiliations:** 1 Department of Plastic Surgery, Kyorin University School of Medicine, Tokyo, Japan; 2 Department of Plastic and Reconstructive Surgery, Graduate School, Tokyo Medical and Dental University, Tokyo, Japan; 3 Department of Parasitology, Kyorin University School of Medicine, Tokyo, Japan

**Keywords:** fibrosis, scarring, fibroblast, heterogeneity, tissue specificity

## Abstract

Clinically, wounds on the face tend to heal with less scarring than those on the trunk, but the causes of this difference have not been clarified. Fibroblasts obtained from different parts of the body are known to show different properties. To investigate whether the characteristic properties of facial and trunk wound healing are caused by differences in local fibroblasts, we comparatively analyzed the functional properties of superficial and deep dermal fibroblasts obtained from the facial and trunk skin of seven individuals, with an emphasis on tendency for fibrosis. Proliferation kinetics and mRNA and protein expression of 11 fibrosis-associated factors were investigated. The proliferation kinetics of facial and trunk fibroblasts were identical, but the expression and production levels of profibrotic factors, such as extracellular matrix, transforming growth factor-β1, and connective tissue growth factor mRNA, were lower in facial fibroblasts when compared with trunk fibro-blasts, while the expression of antifibrotic factors, such as collagenase, basic fibroblast growth factor, and hepatocyte growth factor, showed no clear trends. The differences in functional properties of facial and trunk dermal fibroblasts were consistent with the clinical tendencies of healing of facial and trunk wounds. Thus, the differences between facial and trunk scarring are at least partly related to the intrinsic nature of the local dermal fibroblasts.

## Introduction

Fibroblasts are the most common cells present in connective tissues, where they synthesize extracellular matrix (ECM) and play a critical role in wound healing [1]. Fibroblasts are known to be composed of diverse cell populations and manifest pheno-typic differences in their function, such as ECM production and organization, and production of growth factors and cytokines [2–5]. These differences in properties are notable in cutaneous pathological conditions such as keloid [6], hypertrophic scar [7], scleroderma [8], café au lait macule [9], and neurofibroma [10], and even under physiological conditions, fibroblasts exhibit differences; this is known as fibroblast heterogeneity [3–5]. The functional differences are particularly evident between superficial dermal fibroblasts and deep dermal fibroblasts [3–5,11–15]. Heterogeneity also exists between anatomical locations, as several recent studies have indicated that fibroblasts from different body sites retain positional information and topographic differentiation patterns in the expression of genes in vitro [16,17]. However, there are limited reports of the differences in wound healing-associated functions between dermal fibroblasts based on body sites [18].

Clinically, wounds on the face and trunk show different tendencies for wound scarring [19]. Facial incisional wounds, particularly preauricular incisional wounds, heal with less scarring than similar wounds on the trunk. Factors such as the innate properties of resident cells, thickness and compositional structure of the dermis, perfusing blood flow, and mechanical stresses such as skin tension are thought to be the reasons for such differences [19,20]. However, to our knowledge, no studies have elucidated the specific mechanisms responsible.

In order to clarify whether the characteristic properties of facial and trunk scarring are due to differences in local fibroblasts, functional differences between facial dermal fibroblasts and trunk dermal fibroblasts were investigated, using primary facial superficial dermal fibroblasts (FS), facial deep dermal fibroblasts (FD), trunk superficial dermal fibroblasts (TS), and trunk deep dermal fibroblasts (TD). Thus, cellular proliferation kinetics, and expression and production of 11 fibrosis-associated factors, including representative ECM metabolism-associated factors or cytokines, which are considered to be related to scar fibrosis, were investigated and compared between facial and trunk dermal fibroblasts.

## Materials and Methods

### Cell Isolation and Culture

Facial (preauricular) and trunk (lateral thoracic) skin was obtained during reconstructive surgery from seven healthy donors without antecedent operative invasion to the biopsied site (four females and three males; age, 46.6 ± 14.4 years). Profiles and data description codes are shown in Table 1. The research protocol was approved by the internal review board of our university hospital. Informed consent was obtained from all patients. After resection of subcutaneous tissues, specimens were washed three times in phosphate-buffered saline, and the external and internal surfaces were dermatomed in order to obtain superficial and deep dermal samples, respectively. To avoid cellular selection bias, both samples were incubated with 0.25% trypsin and 0.02% EDTA in phosphate-buffered saline for 16–24 hr at 4°C and the epithelium was separated from the superficial dermal sample. From the separated superficial and deep dermal samples, human fibroblasts were cultured as explants and maintained at 37° C under a 5% CO_2_ atmosphere in fibroblast growth medium (FGM) consisting of Dulbecco's modified Eagle's medium (DMEM) supplemented with 10% fetal calf serum and 0.6 mg/ml glutamine. After about 3 weeks, primary cultures were subcultured. Medium was replaced every 3 days during cell culture.

**Table 1:** Profiles and data description codes of samples

No.	Age	Sex	Proliferation assay	Real-time PCR assay	ELISA (collagen I)	ELISA (cytokines)	Symbol in figures
1	44	M	*	*	*	*	○
2	34	M	*	*	*	*	△
3	60	F	*	*	*	*	□
4	65	M	*	*	*	*	●
5	53	F	*	*	*	*	▲
6	24	F	*	*		*	■
7	46	F	*	*			×

### Proliferation Assay

Cells at the third passage were plated in triplicate in 12-well plates at 1.0 × 10^4^ cells/well in FGM. Medium was replaced every 4 days and the cell number was manually counted at the same time until day 32.

### Quantification of mRNA by Real-Time Polymerase Chain Reaction

Cells at four or five passages were plated in 12-well plates at 1.0×10^4^ 10 cells/well in FGM. On day 4, total RNA was isolated using an RNeasy™ Mini Kit and QIA shredder (both from QIAGEN, Hilden, Germany), followed by reverse transcription using a High Capacity RNA-to-cDNA kit. Wound maturation-associated genes (listed in Table 2) were quantified using real-time polymerase chain reaction (PCR). Reaction mixtures comprised 10 μl of FAST SYBR® Green Master Mix and 1 μl of cDNA sample and RNase-free water with the indicated primer concentrations. Reactions were performed and monitored using the StepOnePlus™ real-time PCR system. All PCR reagents and the PCR system were obtained from Applied Biosystems (Foster City, CA, USA). PCR comprised 40 cycles, consisting of denaturing at 95°C (3 s) and annealing/extension at 60°C (15 s). Primer sequences were designed based on previous studies and were optimized for concentration [8,21-25]. Primers for which amplification efficiency was between 1.95 and 2.05 were employed for the study. Sequences and optimized concentrations for each primer are shown in Table 2. For quantification, expression levels were calculated by the comparative CT method using glyceraldehyde-3-phosphate dehydrogenase (GAPDH) and human acidic ribosomal protein (HARP) as an endogenous reference gene. Preliminary tests confirmed that both the endogenous controls offered similar results. Therefore, we decided to use GAPDH as an internal control throughout the study.

**Table 2:** Primer sequences and optimized concentrations for real-time PCR

Gene	Coding protein	Sequence	Primer concentration [nM]
COL1A1	TypeIcollagen	F:CCCACCAATCACCTGCGTACAGA R: TTCTTGGTCGGTGGGTGACTCTGA	100 100
COL3A1	Type III collagen	F:GAGATGTCTGGAAGCCAGAACCAT R: GATCTCCCTTGGGGCCTTGAGGT	100 100
FN1	Fibronectin	F:GGAGAATTCAAGTGTGACCCTCA R: TGCCACTGTTCTCCTACGTGG	300 300
MMP1	MMP1 (collagenase)	F:TCTGGGGTGTGGTGTCTCA R:GCCTCCCATCATTCTTCAGGTT	300 300
TGFB1	TGF-β1	F:GTTCAAGCAGAGTACACACAGC R: GTATTTCTGGTACAGCTCCACG	300 300
TGFB2	TGF-β2	F:ATGCGGCCTATTGCTTTAGA R: TAAGCTCAGGACCCTGCTGT	200 200
TGFB3	TGF-β3	F:CAGGGAGAAAATCCAGGTCA R: CCTGGAAGGCGTCTAACCAAG	100 100
CTGF	CTGF	F:ACGGCGAGGTCATGAAGAAGAACA R: ACTCTCTGGCTTCATGCCATGTCT	100 100
ASMA	α-smooth muscle actin	F:CCAAGCACTGTCAGGAAT R: AGGCAGTGCTGTCCTCTT	100 100
FGF2	bFGF	F:ATACAGCAGCAGCCTAGCAACTCT R: TTCGGCAACAGCACACAAATCCTG	100 100
HGF	HGF	F:GCAAGTGAATGGAAGTCCTTTA R: CAGAGGGACAAAGGAAAAGAA	100 100
SCF	SCF	F:GCCGCTGTTCGTGCAATAT R: CTGCGATCCAGCACAAACAGT	200 200
HARP	HARP	F:CGCTGCTGAACATGCTCAA R: TGTCGAACACCTGCTGGATG	300 300
GAPDH	GAPDH	F:GAAGGTGAAGGTCGGAGTC R: GAAGATGGTGATGGGATTTC	300 300

### Measurement of ECM and Cytokines by Enzyme-Linked Immunesorbent Assay

Samples for enzyme-linked immunesorbent assay (ELISA) were collected under the same culture conditions as cDNA. Soluble and sediment type I collagens were quantitatively analyzed by ELISA (Human collagen I EIA kit; Applied Cell Biotechnologies, Inc., Yokohama, Kanagawa, Japan) according to the manufacturer's instructions. Transforming growth factor-ß1 (TGF-ß1), transforming growth factor-ß2 (TGF-ß2), and connective tissue growth factor (CTGF) in culture supernatants were also measured by ELISA (Quantikine from R&D Systems (Minneapolis, MN, USA) for TGF-ß1 and TGF-ß2, and Human CTGF ELISA Kit from Cusabio, Inc., Wuhan, Hubei, China, for CTGF), in accordance with the manufacturer's instructions. Levels of each factor were measured using a microplate reader (Power Scan® HT; Dainippon Pharmaceutical, Osaka, Japan). Data are expressed as secreted factors per 1.0×10^4^ cells at the time of harvest.

### Statistical Analyses

Differences in values between groups of cells were analyzed by paired Student's *t*-test. In the proliferation assay, associations between cell number of facial dermal fibroblasts and trunk dermal fibroblasts at confluence (on day 32) were statistically analyzed using Pearson's correlation index. Values of *p* < 0.05 were considered to be statistically significant. Chronological changes in cell number are presented as means ± standard error of the mean, and other data are presented as means ± standard deviation.

## Results

### Cell Morphology and Proliferation

**Figure 1:**
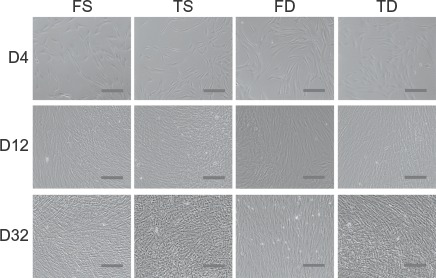
Cell morphology of FS, TS, FD, and TD. Phase contrast microscopic findings for FS, TS, FD, and TD from donor No. 4 at 4,12, and 32 days after cell seeding. Scale bar indicates 100 μm.

**Figure 2:**
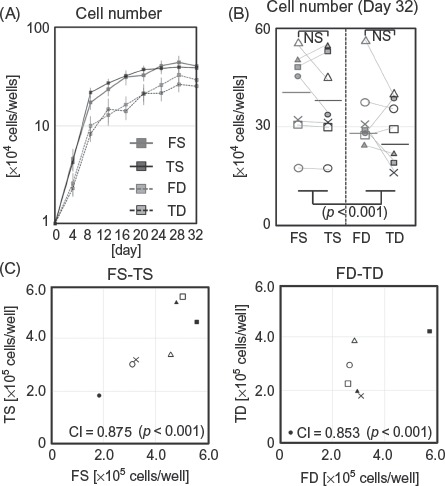
Cell proliferation of FS, TS, FD, and TD. (A) Chronological cell number in four cell fractions is noted (*n* = 7 for each). Error bars indicate SEM. (B) Cell count of FS, TS, FD, and TD on day 32 (*n* = 7 for each). (C) Correlation of saturated cell number between facial and trunk fibroblasts on day 32.

Morphologically, no differences were observed between dermal fibroblasts obtained from the face and trunk, while superficial dermal fibroblasts and deep dermal fibroblasts showed apparent differences regardless of donor site. Superficial dermal fibroblasts were smaller and spindle-shaped when compared with deep dermal fibroblasts, which tended to broadly spread on the surface (Figure 1). In addition, with regard to proliferation kinetics, no differences were observed between facial and trunk dermal fibroblasts, although the cellular density of superficial dermal fibroblasts tended to be higher than that of deep dermal fibroblasts on proliferation assay (Figure 2A), as shown in the comparative description of cell numbers of FS, FD, TS, and TD on day 32 (Figure 2B). Cell numbers at confluency on day 32 demonstrated that for superficial dermal fibroblasts and deep dermal fibroblasts, respectively, cellular density of facial and trunk dermal fibroblasts from the same donor showed significant correlations (Figure 2C).

### mRNA Expression of Fibrosis-Associated Factors

**Figure 3:**
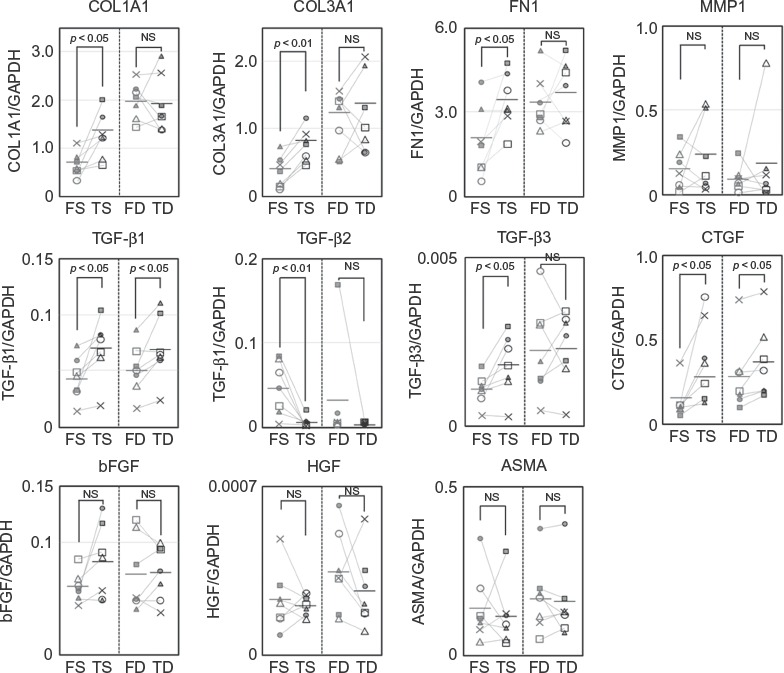
Expression of wound healing-associated factors by FS, TS, FD, and TD. mRNA expression of fibrosis-associated factors by FS, FD, TS, and TD (*n* = 7, each).

In order to investigate the functional differences between facial and trunk dermal fibroblasts, mRNA expression of fibrosis-associated factors in superficial dermal fibroblasts and deep dermal fibroblasts was quantitatively compared using real-time PCR. Among superficial dermal fibroblasts, FS showed lower expression of ECMs such as type I and III collagens, fibronectin, TGF-ß1 and TGF-ß3, and CTGF when compared with TS. On the other hand, expression of TGF-ß2 was higher in FS than in TS. Expression of MMP1, ASMA, bFGF, and HGF showed no clear trends. Among deep dermal fibroblasts, FD showed lower expression of TGF-ß1 and CTGF than TD, while no clear trends were seen for other factors (Figure 3).

### Production of Fibrosis-Associated Factors

In order to further confirm the differences between facial and trunk dermal fibroblasts, protein production of type I collagen, TGF-ß1, TGF-ß2, and CTGF were compared by ELISA.

**Figure 4:**
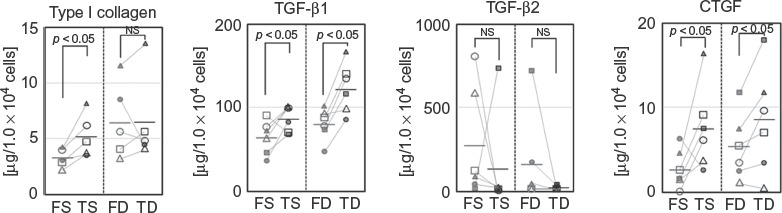
Production of wound healing-associated factors by FS, TS, FD, and TD. Production of type I collagen, TGF-ß1 and TGF-ß2, and CTGF by FS, FD, TS, and TD (for type I collagen, *n* = 5 for each; for others, *n* = 6 for each).

Among superficial dermal fibroblasts, FS showed significantly lower production of type I collagen, TGF-ß1, and CTGF than TS. this was consistent with the results of mRNA expression analysis. In addition, production of TGF-ß2 showed the same trend as for mRNA expression, with higher production in FS than that in TS, although the differences were not significant. Among deep dermal fibroblasts, similar to results of mRNA expression analysis, FD showed lower production of TGF-ß1 and CTGF than TD, and no clear trends were seen for type I collagen and TGF-ß2 (Figure 4).

## Discussion

In our analysis of seven paired FS, FD, TS, and TD samples from the same individuals, facial and trunk dermal fibroblasts obtained from the same layers of dermis showed identical morphology and proliferation kinetics, while differences in depth of origin distinctly affected cell morphology and proliferation kinetics, as indicated in previous reports [11–15]. On the other hand, the cellular density of facial and trunk dermal fibroblasts from the same depth of dermis in the same individuals showed significant, positive correlations. The proliferative capability of superficial and deep dermal fibroblasts is thought not to be influenced by anatomical site, but is specific to donor individuals.

In a subsequent study, mRNA expression of 11 genes considered to be associated with scarring (i.e., fibrosis formation) was investigated between facial and trunk dermal fibroblasts. Type I and III collagens, fibronectin, and collagenase expression were investigated because ECM production causes excess deposition of ECM, and were considered to be a profibrotic factor [8,9,26], while collagenase expression is considered to be an antifibrotic factor [11,27]. Other cytokines, such as TGF-βs, CTGF, bFGF, and HGF, were also investigated because these may be involved in fibrotic processes [11,27–31]. TGF-β1 and its downstream mediator CTGF are known to play particular important roles in hypertrophic scar formation and keloids [6,12,24,30,32] and are therefore considered to be dominant profibrotic factors. TGF-β2 [19,31] and TGF-β3 [19,30,31,33,34] also play important roles in the process of fibrotic scar formation, although their relative dominance during scar development is smaller when compared with TGF-β1 and CTGF, and their effects on scarring were not clearly defined as pro- or antifibrotic. On the other hand, HGF is reported to have antifibrogenic effects in various organs [35,36], and much more recently, bFGF was found to prevent fibrogenesis via activation of HGF secretion from adipose-derived cells and dermal fibroblasts [37]. Therefore, we also investigated the expression of HGF and bFGF as possible antifibrotic factors.

Between facial and trunk dermal fibroblasts, differences in expression and/or production of fibrosis-associated factors have been noted. In particular, it was noted that expression and production of TGF-β1 and CTGF, known as one of the most potent fibrosis-inducing factors [6,12,24,30,32], were lower in facial fibroblasts than in trunk fibroblasts. Moreover, with regard to superficial dermal fibroblasts, facial fibroblasts showed lower expression and/or production of ECMs than trunk fibroblasts. This indicates that facial dermal fibroblasts are intrinsically less fibrotic than trunk dermal fibroblasts, although the reasons for the differences in TGF-β2 and TGF-β3 expressions in fibrosis are unclear.

Our study demonstrated that facial and trunk dermal fibroblasts in the superficial and deep dermis possess identical proliferative capacity, but that facial dermal fibroblasts show lower fibrotic activity in mRNA expression and protein production analyses. We believe that these functional differences in local dermal fibroblasts are at least partly responsible for the clinically observed differences in scarring in the face and trunk. (Facial wounds tend to heal with less scarring than trunk wounds.)

As noted by Chang et al. in a genome-wide mRNA expression analysis using a microarray, fibroblasts obtained from various body sites displayed distinct and characteristic transcriptional patterns, particularly with regard to HOX genes established during embryogenesis, and fibroblasts at different locations in the body should be considered as distinct differentiated cell types [16]. During the developmental process, the dermal component is generally derived from lateral plate mesoderm and somite [38], however, the dermis of the face and ventral neck area is specifically differentiated from neural crest cells via formation of mesoecto-derm [38,39]. We believe that these developmental differences are the cause of the functional differences observed in our study. Future studies should aim to clarify more fundamental differences between facial and dermal fibroblasts, similar to the work of Yamaguchi et al., which revealed that the physiological differences in melanin pigment between palmoplantar and nonpalmoplantar skin are associated with elevated expression of dickkopf-1, an inhibitor of the canonical Wnt signaling pathway which is also associated with developmental processes [18]. Further clarification of key factors in the anatomical differences in the scarring properties of fibroblasts may contribute to future therapeutic intervention for problematic wound scarring, such as conspicuous scars resulting from excess scar formation, hypertrophic scarring, or keloids.

## Conclusions

Our study demonstrated that facial and trunk dermal fibroblasts in the superficial and deep dermis possess identical proliferative capacity, but that facial dermal fibroblasts show lower fibrotic activity in mRNA expression and protein production analyses. The differences in functional properties of facial and trunk dermal fibroblasts were consistent with the clinical healing tendencies of facial and trunk wounds. Thus, the differences between facial and trunk scarring are, in part, related to the intrinsic nature of the local dermal fibroblasts.
